# Dermal absorption of aromatic amines in workers with different skin lesions: a report on 4 cases

**DOI:** 10.1186/1745-6673-1-17

**Published:** 2006-07-19

**Authors:** Gintautas Korinth, Tobias Weiss, Jürgen Angerer, Hans Drexler

**Affiliations:** 1 Institute and Out-Patient Clinic of Occupational, Social and Environmental Medicine, University of Erlangen-Nuremberg, Schillerstrasse 25/29, D-91054 Erlangen, Germany; 2 Research Institute for Occupational Medicine of the Ruhr University of Bochum (BGFA), Bürkle-de-la-Camp Platz 1, D-44789 Bochum, Germany

## Abstract

There are only few studies about the relationship of skin lesions and the percutaneous uptake of hazardous substances in exposed workers. Several aromatic amines are well known carcinogens for humans and/or animals. This case report emphasizes the relevance of dermal absorption of the aromatic amine *ortho*-toluidine considering four workers with different skin status (healthy, erythematous and burned skin as well as dishydrotic eczema) during the vulcanisation process of rubber products in a components supplier plant for automobile industry. The results of our case report show that dermal absorption of *o*-toluidine through damaged epidermal barrier is significantly higher than through healthy skin.

## Background

The exposure to aromatic amines (AA) continues to be a serious problem. Bladder cancer caused by exposure to AA is a common occupational cancer. The greatest problem from the point of view of occupational hygiene is, in spite of the standard technical protection measures, that AA are well absorbed through the skin.

AA, respectively the intermediates of AA, are not substitutable in various industries until now. *Ortho*-toluidine (*o*-toluidine) is classified by the International Agency for Research on Cancer (IARC) as probably carcinogenic to humans [1]. In Germany this compound is considered to be carcinogenic for man [2].

In automobile industry *o*-toluidine is contained in di-ortho-tolylguanidine used as accelerator for the vulcanisation of rubber products. In the present case report we evaluate the dermal absorption of *o*-toluidine in workers who performed vulcanisation of rubber articles.

## Methods

### Study population

In a components supplier plant for the automobile industry we examined all workers (n = 4, male, response rate: 100%) involved in vulcanising hydraulic rubber articles. During this process the workers were exposed by inhalation and/or dermal contact to *o*-toluidine released from vulcanisation accelerators. We asked the workers to report on general workplace conditions, private and occupational risk factors, skin complaints, history of skin diseases and the use of personal protective equipment (breathing protection masks, gloves, skin barrier and skin care creams) by means of a standardized, self-administered questionnaire.

### Clinical examination of skin

A dermatologically trained physician screened the skin of the workers for lesions in accordance with a recently published study [3]. Lesions considered included erythema, scaling and other pathological findings such as fissures, vesicles or callosities, categorized also according to anatomical site. The classification of erythema and skin scaling was performed immediately after clinical examination. Hence, the observer was blind to type and intensity of exposure when evaluating the skin.

### External exposure and biological monitoring

We measured the concentration of *o*-toluidine in the workplace air by personal air monitoring (NIOSH method No. 2017) [4]. Internal exposure in workers was determined by analysis of *o*-toluidine in post-shift urine using gas chromatography and mass selective detection with negative chemical ionisation [5,6]. As a rule, this is the optimal time point for urine sampling to assess the exposure over a working day [2]. For the direct comparison of the internal exposure in workers related to the external exposure we used the quotient RIE (relative internal exposure) as described by Drexler et al. [7]:



We regard this quotient as a measure of the internal exposure related to the individual exposure. On this basis, we were able to compare the individual relationships of internal to external exposure. Additionally we assessed the influence of smoking on internal exposure by measurement of cotinine in the urine of the workers. In view of the explanatory nature of our data, we used the RIE index to compare the values of external and internal exposure between the workers.

## Results and discussion

The results of personal air and biological monitoring to *o*-toluidine are presented in Table 1. Over the whole shift (8 hours), the workers were exposed to AA by inhalation and by dermal contact from the gaseous phase. The German threshold limit value for *o*-toluidine in the air (500 μg/m^3^) was not exceeded [2]. However, the concentration of *o*-toluidine in the workplace air was rather high as indicated by a factor of at least 25 compared to values found in indoor and outdoor air [8,9]. Cotinine values in urine showed that workers no. 3 and 4 were non-smokers, probably not even exposed by passive smoking. Therefore, comparing the urine values presented in our case report with the background exposure level of the German general population (range: <0.05 – 3.1 μg/l, median: 0.12 μg/l) we can assume that the internal exposure to *o*-toluidine in all workers resulted primarily from occupational exposure [5].

**Table 1:** Values from personal air and biological monitoring.

Variables	Worker no. 1	Worker no. 2	Worker no. 3	Worker no. 4
Skin condition of hands	Healthy skin	Mild erythema	Burns on hands	Dyshidrotic eczema
*o*-Toluidine in the air (μg/m^3^)	58.29	93.93	32.73	26.63
*o*-Toluidine in urine (μg/l)	74.83	242.88	64.36	54.65
Cotinine in urine (μg/l)	4471 μg/L (= smoker)	741 μg/L (= smoker)	< 5 μg/L (= non-smoker)	< 5 μg/L (= non-smoker)

The skin status as well as skin protection and care can affect the uptake of AA. None of workers was equipped with breathing protection masks against inhalative uptake of AA. All four workers wore thick cloth gloves during contact to the vulcanised rubber tubes. The dermal contact of the hands to AA was very similar for all workers and existed with short interruptions over the whole work shift. During the wearing of cloth gloves (210 – 240 minutes) the hands were occluded also in a wet environment. The cloth gloves were replaced during the shift 7 – 12 times after being wetted by the work. We did not observe atopic skin diathesis in the workers. One worker (no. 1) had healthy skin. However, the three other workers had skin lesions of different kind and severity. While the skin of worker no. 2 was affected only by a slight erythema on the hands, in worker no. 3 we observed several sites on hands with burns (though covered by scab or visible as redness) caused by accidental contacts to hot (about 180°C) pipes serving to form rubber tubes during the vulcanisation process. The most severe skin lesions were observed in worker no. 4 suffering from dyshidrotic eczema for more than 16 years and moderate erythematous lesions on hands and forearms.

**Figure 1:**
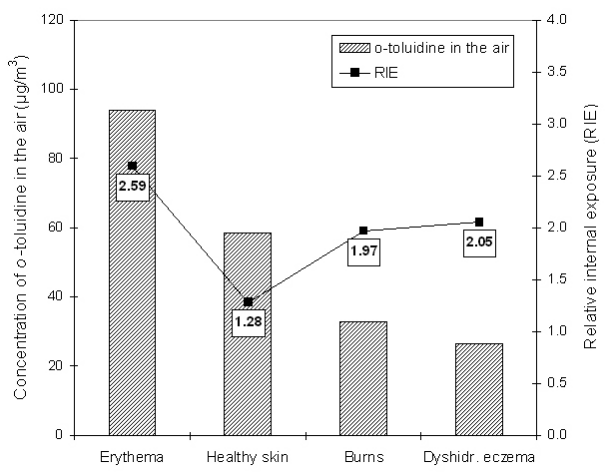
Relationship between the concentration of *o*-toluidine in ambient air at the workplace and the relative internal exposure (RIE).

The results show that the RIE depends on skin condition of the workers (Fig. 1). The external inhalative exposure to *o*-toluidine in the worker suffering from dyshidrotic eczema was the lowest of all workers (by a factor of 3.5 lower than in highest exposed worker), but he showed the second highest RIE (Fig. 1). The external inhalative exposure to *o*-toluidine in the worker with healthy skin was the second highest. However, he showed the lowest RIE of all workers, despite the fact that he was additionally exposed to AA from heavy smoking (Table 1). Worker no. 2 (mild erythema) was the highest exposed worker by inhalation (at the factor of 3.5 higher than the worker with dyshidrotic eczema). However, this high air exposure did not lead to a proportional increase of RIE. These slight erythematous changes in the worker no. 2 do not seem to have a significant effect on the skin barrier preventing the dermal uptake of the lipophilic *o*-toluidine.

It is well known that damaged epidermal barrier leads to a higher dermal absorption of chemicals [10,11]. Internal exposure to solvents miscible in water is higher in workers with skin lesions than in workers with healthy skin [3,7]. Moreover, recently in vitro experiments showed that *o*-toluidine penetrates through human skin to a high extent. These experiments were based on a finite exposure dose in order to mimic occupationally relevant situations [12]. Therefore, due to the low vapour pressure, the internal exposure of workers to *o*-toluidine in the present study should result predominantly from dermal absorption.

In our study, a relationship between skin lesions and the internal exposure to the AA *o*-toluidine was established. The uptake of *o*-toluidine was relatively higher in workers with damaged epidermal barrier such as skin burns, particularly with dyshidrotic eczema, than in workers with healthy or slight erythematous skin. A proportional relationship between the airborne levels of *o*-toluidine and the difference of RIE (Fig. 1) emphasize the importance of its dermal absorption in workers. These findings are in accordance with our previous studies with a glycol ether and carbon disulfide [3,7]. From an occupational medical point of view the higher uptake of chemicals in workers with impaired skin barrier cannot be ignored. Gloves (the material itself or the frequency of replacement) and skin creams do not seem to be sufficient to achieve an efficient protection. However, due to the small study group, the impact of various occupational exposure factors on dermal absorption of *o*-toluidine cannot be conclusively assessed. Solely the worker with dyshidrotic eczema used skin barrier creams at the workplace. He and the worker with healthy skin used skin care creams in the plant. The protective effects of skin creams to reduce the dermal absorption were not evident in our study group. On the contrary, there are hints that barrier creams might enhance the dermal absorption [13].

In our study, the workers with the highest internal exposure (RIE) coincidentally had the lowest airborne exposure. From the perspective of preventive occupational medicine, we have to assume the opposite to be the case: occupational exposure scenarios that workers with severe skin lesions have a high inhalative exposure. This would lead to a disproportionately high *o*-toluidine uptake that is not sufficiently reflected by sole determinations of *o*-toluidine in air. Therefore we recommend to perform biological monitoring for workers with skin diseases. The assessment of haemoglobin adducts – a long-term parameter reflecting the cumulative internal exposure of about the last 4 months in accordance with the lifetime of the erythrocytes – is a powerful tool for the surveillance of occupationally exposed workers. Only the approach of biological monitoring can assess the uptake of hazardous substances by all routes.

## Conclusion

Our case report shows that the internal exposure to AA increases in workers with impaired epidermal barrier. When the dermal contact to hazardous substances at workplaces cannot be avoided, respectively a sufficient exposure assessment to prevent the dermal uptake is not practicable, biological monitoring of workers can help to monitor the total body burden.

## Declaration of competing interests

The author(s) declare that they have no competing interests.

## Abbreviations

AA; aromatic amines

IARC; International Agency for Research on Cancer

RIE; relative internal exposure

## Authors' contributions

GK and TW were the principal investigators. GK examined the workers and drafted the manuscript. TW and JA were responsible for analyses of personal air and biological monitoring. TW, JA and HD revised critically the manuscript. All authors read and approved the final manuscript.
